# Association between Sarcopenic Obesity and Changes in Skeletal Muscle Mass and Quality in Patients with Stroke Who Undergo Convalescent Rehabilitation

**DOI:** 10.31662/jmaj.2024-0370

**Published:** 2025-03-21

**Authors:** Ryo Shiraishi, Nami Shiraishi, Haruhiko Sato, Takuya Tanaka, Keita Shimizu, Kota Okumura, Kou Suzuki, Takahiro Ogawa

**Affiliations:** 1 Clinical Education and Research Center, Chuzan Hospital, Okinawa, Japan; 2 Department of Clinical Research and Quality Management, Graduate School of Medicine, University of The Ryukyus, Okinawa, Japan; 3 Department of Rehabilitation Medicine, Aichi Medical University, Nagakute, Japan

**Keywords:** stroke, sarcopenia, skeletal muscle mass, phase angle, rehabilitation

## Abstract

**Introduction:**

Sarcopenic obesity substantially affects the recovery of physical function in patients with stroke. However, few studies have investigated the relationship between changes in skeletal muscle mass (SMM) and skeletal muscle quality (SMQ) and sarcopenic obesity diagnosed using the Japanese Working Group on Sarcopenic Obesity (JWGS) diagnostic criteria in patients with stroke who undergo rehabilitation. Therefore, this study aimed to investigate the relationship between sarcopenic obesity and changes in SMM and SMQ in patients with stroke who undergo rehabilitation.

**Methods:**

Patients with stroke admitted to a rehabilitation ward in a single center in Japan were enrolled in this retrospective cohort study. The inclusion criteria were age 40-75 years and hospitalization for rehabilitation therapy due to stroke. The exclusion criteria were length of hospital stay <14 days and missing clinical data. Data were collected from medical records. Classification of sarcopenic obesity was based on the JWGS diagnostic criteria. The outcomes were the change in SMM and phase angle (PhA) from admission to discharge. Multiple regression analysis was used to investigate the relationship between sarcopenic obesity and changes in SMM and PhA after adjustment for confounding factors.

**Results:**

A total of 173 patients were analyzed. 8 patients (3 male and 5 female) were diagnosed with sarcopenic obesity using the JWGS criteria. Multiple regression analysis revealed that sarcopenic obesity was negatively associated with changes in SMM (β: −0.281, 95% confidence interval [CI]: −0.449 to −0.113, p < 0.001) and PhA (β: −0.189, 95% CI: −0.367 to −0.010, p = 0.038).

**Conclusions:**

Sarcopenic obesity is negatively associated with changes in SMM and SMQ in patients with stroke who undergo rehabilitation, highlighting the importance of evaluating sarcopenic obesity in patients with stroke from an early stage.

## Introduction

Sarcopenic obesity is a condition in which sarcopenia and obesity coexist ^(1), (2)^. To date, definitions of sarcopenic obesity reported by the European Society for Clinical Nutrition and Metabolism and the European Association for the Study of Obesity have been used to diagnose the condition ^(3)^. Although these definitions do not necessarily apply to all populations, they have historically been used for diagnosis in different groups. Therefore, the Japanese Working Group on Sarcopenic Obesity (JWGS) has reported new diagnostic criteria for sarcopenic obesity that can be applied to Asian populations ^(4)^.

Previous reports in Asian patients with stroke have shown that sarcopenic obesity is associated with impaired oral conditions ^(5)^. Moreover, sarcopenic obesity is negatively associated with the phase angle (PhA) ^(6)^. Furthermore, cross-sectional studies have reported that it is associated with a decrease in activities of daily living (ADLs) ^(7)^. These reports also suggest that sarcopenic obesity is an important problem in patients with stroke.

A review in patients with stroke revealed that skeletal muscle mass (SMM) and skeletal muscle quality (SMQ) are important clinical factors ^(8), (9)^. A decrease in SMM in patients with stroke limits the recovery of physical function ^(10)^. In addition, a reduction in SMM is associated with a decrease in ADL ^(5)^. Furthermore, a combination of low SMM and SMQ was reported to increase the incidence of infectious pneumonia after a stroke ^(11)^. These findings suggest that the SMM and SMQ in patients with stroke are important factors for the recovery of physical function and ADL, and for providing appropriate care.

To the best of our knowledge, no previous studies have investigated the relationship between changes in SMM and SMQ and sarcopenic obesity diagnosed using the JWGS criteria in patients with stroke who undergo rehabilitation. We considered that clarifying these relationships would contribute to advancing the academic understanding of the condition of patients with stroke. Therefore, this study aimed to investigate the relationship between sarcopenic obesity and changes in SMM and SMQ in patients with stroke who undergo rehabilitation.

## Materials and Methods

### Study design

This was a single-center, retrospective cohort study in patients admitted to a rehabilitation ward in Japan between April 2020 and March 2024.

### Ethics approval

This study protocol was approved by the Ethics Review Board of Chuzan Hospital (approval number: 24-11) and was conducted with careful attention to handling personal information. Because of the study’s retrospective nature, an opt-out procedure was used to provide all patients with the option of excluding their data from the analysis ^(12)^. All experimental procedures were performed according to the principles of the Declaration of Helsinki (revised October 2013).

### Participants

The inclusion criteria were patients aged 40-75 years who had been hospitalized because they required rehabilitation therapy owing to stroke. The exclusion criteria were patients with a length of hospital stay of <14 days and those with missing clinical data on admission and discharge, and on the diagnosis of sarcopenic obesity.

### Procedures

#### Data collection

Data including age, sex, body mass index (BMI), stroke type, stroke severity (National Institutes of Health Stroke Scale [NIHSS] score), Mini Nutritional Assessment-Short Form (MNA-SF) score, days from onset to admission to a rehabilitation ward, length of hospital stay, rehabilitation volume, energy intake, and protein intake were collected from medical records. Stroke severity was evaluated using the NIHSS, in which scores range from 0 to 42, with higher scores indicating greater neurological severity ^(13)^. A registered physical therapist assessed the NIHSS score within 1 week of admission. The MNA-SF was used as the nutritional screening tool and was administered by a registered dietitian on admission. The MNA-SF has been reported to be a useful tool for screening nutritional status ^(14)^. Energy and protein intakes were averaged for the week before discharge and retrospectively examined using data recorded by nurses and registered dietitians. Daily energy and protein intakes per kilogram were calculated by dividing the values by the patient’s current body weight. The duration of the rehabilitation program, which included standing, walking, stretching, strength training, and ADL training, was approximately 60-180 min/day. These rehabilitation programs were tailored to the patient’s condition by adjusting the intensity of training.

#### Diagnosis of sarcopenic obesity

Sarcopenic obesity was diagnosed through a two-step process using the algorithm reported by the JWGS ^(4)^. First, sarcopenia and obesity were evaluated through screening. Calf circumference (male: <34 cm, female: <33 cm) was used to evaluate sarcopenia, and BMI (≥25 kg/m^2^) was used to evaluate obesity. The circumference of the calf was measured within 1 week of admission. The calf circumference was measured in the supine position, and the maximum value of the measurement value on either the paretic side or non-paretic side was adopted. Second, patients who were suspected of having sarcopenic obesity during screening were diagnosed with sarcopenic obesity on the basis of a combination of grip strength (male: <28 kg, female: <18 kg) and limb SMM corrected for BMI (male: <0.789 kg/BMI, female: <0.512 kg/BMI) and body fat percentage (male: ≥20%, female: ≥30%). Handgrip strength was measured within 1 week of admission, using a Smedley hand dynamometer (Grip-D, Takei Kiki Kogyo, Niigata, Japan), and the maximum value of the measurement value on either the paretic side or non-paretic side was adopted. In this study, the patients were defined as the sarcopenic obesity group and no sarcopenic obesity group on the basis of the diagnosis of sarcopenic obesity.

#### Limb SMM corrected for BMI

Limb SMM, which is a measure of overall SMM, was measured using the direct segmental multifrequency (DSM) method-bioelectrical impedance analysis (BIA) with the InBody S10 Analyzer (InBody Japan, Tokyo, Japan). The DSM-BIA uses an 8-point tactile electrode system for measurements at six frequencies (1, 5, 50, 250, 500, and 1000 kHz) ^(15)^. Measurements using the InBody S10 Analyzer are taken after the patient rests supinely for 15 minutes, with electrodes attached to the thumbs. Consequently, it can be used without placing any burden on the patient, which is a major advantage. The data measured by the dietitian within 7 days of admission and before discharge were retrospectively analyzed. Limb SMM was calculated by dividing SMM by BMI. In this study, the SMM change (SMM at discharge − SMM at admission) was calculated as the primary outcome.

#### PhA

The PhA, which is a measure of SMQ, was evaluated using BIA. The measurement device used was the InBody S10 Analyzer. The PhA was calculated using resistance (R) and reactance (Xc; measured at 50 kHz) in the following formula: PhA (degrees) = arctangent (Xc/R) × (180/π). The measurement of PhA is highly reliable and accurate, and the error due to intra-day fluctuations in measurement is small ^(16), (17)^. In this study, the change in PhA (PhA at discharge − PhA at admission) was calculated as the second outcome.

#### Sample size calculation

The sample size was calculated using G*power software 3.1 ver. 3.1.9.6 (Heinrich Heine University Düsseldorf, Germany) ^(18)^, a free power analysis program for the implementation of various statistical tests for performing sample size calculations ^(19)^. Assuming the multiple regression model has the nine explanatory variables for which the standard partial regression coefficient should be estimated, and given that a moderate effect size (f^2^ = 0.15) ^(20)^ is obtained, we calculated a total sample size of 114 patients for this study at an α error of 0.05 and power of 0.8. Therefore, data were collected from >114 participants.

### Statistical analyses

All continuous variables were tested for normality using the Kolmogorov-Smirnov test. Parametrically and nonparametrically distributed quantitative variables are presented as mean (standard deviation) and median with interquartile range, respectively. Qualitative variables are expressed as frequencies. t-Tests or Mann-Whitney U-tests were used for quantitative variables, and chi-square tests were used for categorical variables.

Multiple regression analysis was performed to investigate the relationship between sarcopenic obesity and changes in SMM and PhA. Covariates in the multiple regression analysis included variables that were reported to be related to SMM and PhA in previous studies or variables that were deemed clinically related ^(21), (22), (23)^. The selection of covariates was adjusted on the basis of sample size and multicollinearity. The covariates were age, sex, NIHSS at admission, MNA-SF at admission, length of hospital stay, protein intake, energy intake, and rehabilitation volume, in addition to sarcopenic obesity, sarcopenia, and obesity. Furthermore, we confirmed that there was no multicollinearity because the variance inflation factor (VIF) was <3. Statistical analysis was performed using JMP^Ⓡ^17 (SAS Institute Inc., Cary, NC, USA), and the significance level was set at 5%.

## Results

**Figure 1.**
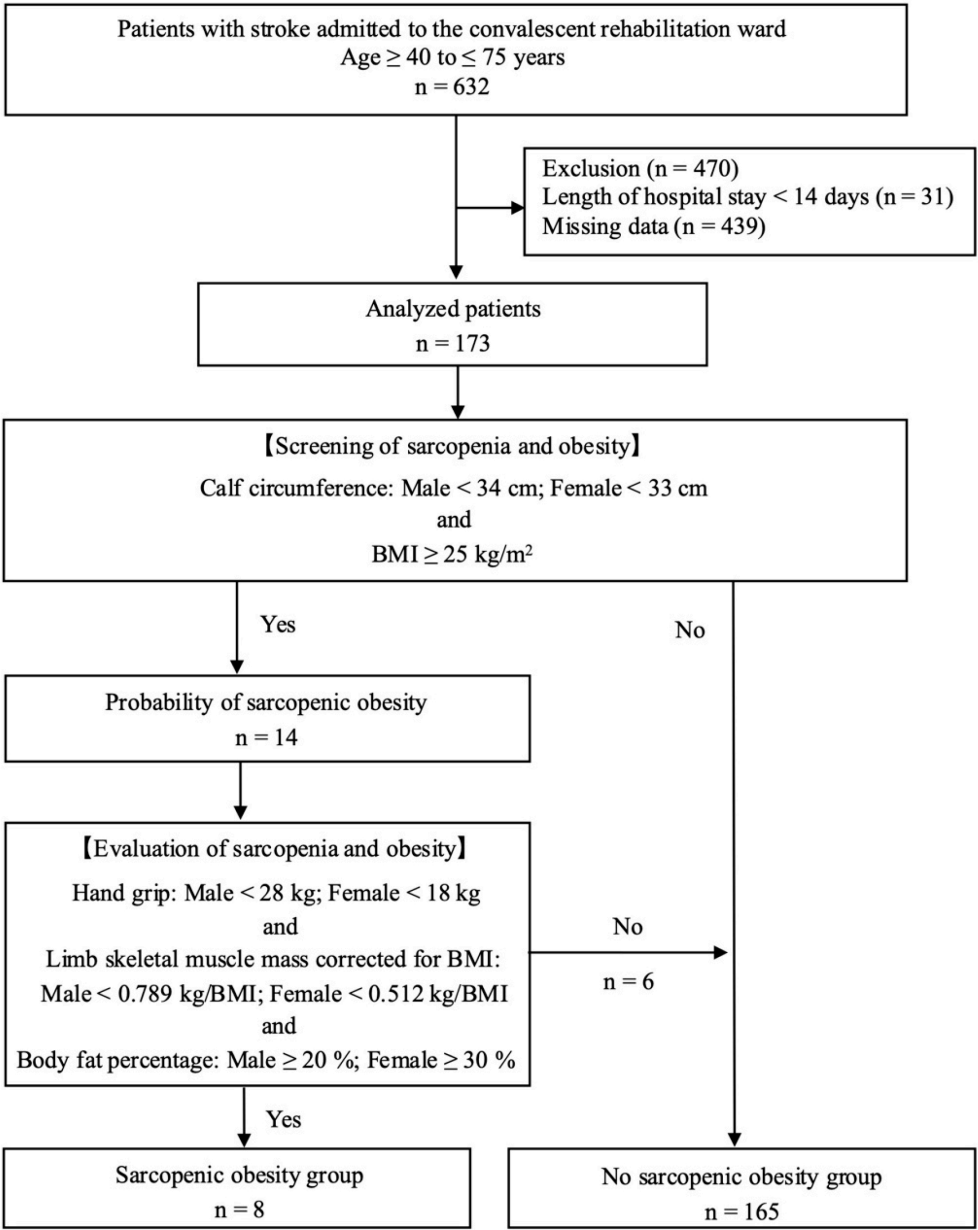
Flowchart of the study BMI: body mass index.

A total of 632 patients aged 40-75 years were hospitalized in the stroke convalescent rehabilitation ward during the survey period, but 470 were excluded owing to a hospital stay of <14 days (n = 31) or missing data (n = 439). Subsequently, 173 patients were included in the analysis (Figure 1).

Table 1 shows the demographic characteristics and clinical data. The median age was 67 [59-71] years, and eight patients (4.5%) were diagnosed with sarcopenic obesity. The sarcopenic obesity group exhibited significant differences in energy intake, SMM at discharge, PhA, change in SMM, and PhA.

**Table 1.** Demographic Characteristics and Clinical Data.

	Overall	Group with sarcopenic obesity	Group without sarcopenic obesity	p Value
	(N = 173)	(n = 8)	(n = 165)	
On admission				
Age, years	67 [59-71]	69 [66-70]	66 [59-71]	0.597
Sex, n (%)				0.264
Male	108 (62)	3 (37)	105 (64)	
Female	65 (38)	5 (63)	60 (36)	
Stroke type, n (%)				0.286
Brain infarction	104 (60)	4 (50)	100 (61)	
Brain hemorrhage	49 (28)	4 (50)	45 (27)	
Subarachnoid hemorrhage	20 (12)	0 (0)	20 (12)	
Sarcopenia, n (%)	106 (61)	8 (100)	98 (59)	0.055
Obesity, n (%)	61 (35)	8 (100)	53 (32)	<0.001
NIHSS score, points	3 [1-5]	5 [4-5]	3 [1-5]	0.166
MNA-SF score, points	8 [7-10]	9 [7-10]	8 [7-10]	0.070
PhA, degrees	4.5 [3.9-5.0]	4.1 [4.0-4.2]	4.5 [3.9-5.1]	0.120
Days from onset to admission to rehabilitation wards, days	17 [11-27]	23 [15-26]	17 [11-27]	0.445
At discharge				
Length of hospital stay, days	64 [39-104]	67.5 [33.2-86.0]	64 [40-105]	0.742
Protein intake, g/kg/day	1.1 [1.0-1.2]	1.0 [1.0-1.1]	1.1 [1.0-1.2]	0.366
Energy intake, kcal/kg/day	29.6 [23.5-37.2]	22.8 [19.7-28.4]	30.4 [24.3-37.4]	0.012
Rehabilitation volume, min/day	143.4 [131.8-158.1]	144.5 [127.4-159.2]	143.4 [132.1-158.1]	0.977
SMM, kg/BMI	0.745 [0.597-0.853]	0.400 [0.367-0.553]	0.755 [0.611-0.859]	<0.001
PhA, degrees	4.6 [4.0-5.2]	4.1 [3.8-4.2]	4.7 [4.1-5.2]	0.013
Changes in SMM, kg/BMI	0.021 ± 0.030	− 0.003 ± 0.011	0.022 ± 0.030	0.022
Changes in PhA, degrees	0.1 ± 0.3	− 0.1 ± 0.3	0.1 ± 0.3	0.011

Parametrically and nonparametrically distributed quantitative variables are presented as mean ± standard deviation and median with interquartile range, respectively. Qualitative variables are expressed as frequencies. BMI: body mass index; MNA-SF: mini nutritional assessment-short form; NIHSS: National Institutes of Health Stroke Scale; PhA: phase angle; SMM: skeletal muscle mass.

Table 2 lists the characteristics of the sarcopenic obesity group on admission. This group showed significant differences in calf circumference and BMI compared with the no sarcopenic obesity group. Moreover, significant differences in grip strength, SMM, and body fat percentage were observed in the evaluation of sarcopenia and obesity.

**Table 2.** Baseline Characteristics of Sarcopenic Obesity Diagnosis in Japan.

	Overall	Sarcopenic obesity group	No sarcopenic obesity group	p Value
	(N = 173)	(n = 8)	(n = 165)	
Screening for sarcopenia				
Calf circumference, cm	32.5 [30.0-35.5]	30.5 [25.9-31.7]	32.5 [30.0-35.6]	0.031
Screening for obesity				
BMI, kg/m^2^	23.2 [21.1-27.0]	26.9 [26.6-28.9]	23.1 [21.0-27.0]	0.007
Evaluation of sarcopenia				
Hand grip, kg	24.4 [18.0-32.9]	14.3 [12.0-16.4]	25.4 [18.4-33.4]	0.002
SMM, kg/BMI	0.719 [0.567-0.836]	0.405 [0.373-0.557]	0.731 [0.588-0.836]	0.002
Evaluation of obesity				
Body fat percentage, %	29.7 [23.4-38.1]	48.4 [42.7-51.3]	29.5 [23.4-36.5]	<0.001

Parametrically and nonparametrically distributed quantitative variables are presented as mean ± standard deviation and median with interquartile range, respectively. BMI: body mass index; SMM: skeletal muscle mass.

Table 3 lists the results of the multiple regression analysis using changes in SMM and PhA as outcome variables. Sarcopenic obesity was negatively associated with changes in SMM (β: −0.281, 95% confidence interval [CI]: −0.449 to −0.113, p < 0.001) and changes in PhA (β: −0.189, 95% CI: −0.367 to −0.010, p = 0.038). VIF was confirmed to be <3.

**Table 3.** Multiple Regression Analysis with Changes in SMM and PhA.

	Changes in SMM			Changes in PhA		
	β (95% CI)	p Value	VIF	β (95% CI)	p Value	VIF
Sarcopenic obesity^a^	−0.281 (−0.449 to −0.113)	<0.001	1.44	−0.189 (−0.367 to −0.010)	0.038	1.44
Sarcopenia^b^	0.164 (−0.038 to 0.366)	0.111	2.08	0.058 (−0.156 to 0.272)	0.593	2.08
Obesity^c^	0.264 (0.060 to 0.468)	0.120	2.12	0.012 (−0.204 to 0.228)	0.913	2.12
Age	0.015 (−0.140 to 0.170)	0.852	1.22	0.107 (−0.058 to 0.271)	0.202	1.22
Sex^d^	−0.102 (−0.273 to 0.070)	0.244	1.50	−0.146 (−0.328 to 0.035)	0.114	1.51
NIHSS score on admission	−0.015 (−0.172 to 0.142)	0.852	1.25	0.006 (−0.160 to 0.172)	0.945	1.25
MNA-SF score on admission	−0.028 (−0.189 to 0.133)	0.732	1.33	−0.060 (−0.231 to 0.111)	0.490	1.32
Length of hospital stay	0.320 (0.157-0.484)	< 0.001	1.36	0.040 (−0.133 to 0.213)	0.646	1.36
Protein intake	−0.085 (−0.227 to 0.057)	0.241	1.03	−0.032 (−0.183 to 0.118)	0.674	1.03
Energy intake	−0.076 (−0.270 to −0.119)	0.444	1.93	0.008 (−0.198 to 0.214)	0.941	1.93
Rehabilitation volume	0.078 (−0.064 to 0.220)	0.281	1.03	−0.011 (−0.162 to 0.139)	0.885	1.02

CI: confidence interval; MNA-SF: Mini Nutritional Assessment-Short Form; NIHSS: National Institutes of Health Stroke Scale; PhA: phase angle; SMM: skeletal muscle mass skeletal muscle mass; VIF: variance inflation factor. ^a^Sarcopenic obesity: No sarcopenic obesity and sarcopenic obesity are coded as 0 and 1, respectively. ^b^Sarcopenia: No sarcopenia and sarcopenia are coded as 0 and 1, respectively. ^c^Obesity: No obesity and obesity are coded as 0 and 1, respectively. ^d^Sex: Male and female are coded as 0 and 1, respectively.

## Discussion

This study examined the relationship between sarcopenic obesity diagnosed using the JWGS criteria and changes in SMM and SMQ in patients with stroke who undergo rehabilitation. The results revealed that in patients with stroke, sarcopenic obesity is negatively associated with changes in SMM and SMQ.

Sarcopenic obesity is a condition in which sarcopenia and obesity coexist, and studies have reported on the prevalence of this condition in patients with stroke ^(24), (25)^. In this study, 4.5% of patients with stroke who underwent rehabilitation had sarcopenic obesity. These results support those of a study that surveyed patients with stroke in Asia ^(24), (25)^. In previous studies, evidence on sarcopenia, obesity, and skeletal muscle changes has been reported separately in patients with stroke ^(8), (26), (27)^. However, to the best of our knowledge, no studies have investigated the relationship between sarcopenic obesity diagnosed using the JWGS criteria and changes in SMM and SMQ; moreover, clinical findings are often unclear.

Skeletal muscle function is known to decrease significantly when obesity is combined with age-related sarcopenia ^(28), (29)^. Therefore, risk factors for both sarcopenia and obesity may affect changes in SMM and SMQ. First, sarcopenic obesity may be associated with changes in SMM, and the inhibitory effect of myostatin on skeletal muscle may be a contributing factor ^(30)^. Previous studies in patients with obesity have shown that hyperinsulinemia caused by obesity enhances the inhibitory effect of myostatin and that this is related to a decrease in SMM ^(31)^. In this study, patients with sarcopenic obesity also had a high BMI and body fat percentage. Therefore, it is possible that these patients had hyperinsulinemia. In addition, previous evidence indicates that obesity can affect skeletal muscle, causing ectopic fat infiltration into or between muscle fibers ^(32)^. Furthermore, obesity causes a decrease in skeletal muscle perfusion and nutrient supply ^(32)^. Considering that these findings directly affect the decrease in skeletal muscle function, it is possible that obesity also affects the qualitative elements of skeletal muscle. However, this study was not designed to test a pathophysiological hypothesis. Therefore, the detailed mechanisms, including pathophysiology, underlying the relationship between sarcopenic obesity and changes in SMM and SMQ in patients with stroke need to be investigated in future research. In this study, in addition to sarcopenic obesity, the length of hospital stay was also positively associated with changes in SMM. In general, patients with severe stroke require longer hospital stays, which likely negatively influences outcomes. However, our results revealed the opposite relationship. Previous evidence indicates that patients with stroke who have low SMM require a sufficient length of hospital stay to increase muscle mass ^(33)^. Furthermore, sufficient rehabilitation time is required for changes in SMM ^(34)^. This evidence also suggests that longer-term rehabilitation may be necessary to change the SMM of patients with stroke who undergo rehabilitation.

Second, chronic inflammation caused by sarcopenic obesity may be associated with changes in SMM and SMQ. Chronic inflammation is a common pathophysiology of sarcopenia and obesity ^(35)^. Sarcopenia, which is associated with aging, has been shown to cause chronic inflammation involving high levels of inflammatory mediators ^(36)^. Moreover, it has been indicated that when obesity develops, inflammatory cytokines produced by immune and mast cells in adipose tissue cause chronic inflammation throughout the body ^(37)^. This common chronic inflammatory response may act directly on skeletal muscle, promoting catabolic effects, such as the breakdown of muscle proteins, and reducing the function of muscle cells (muscle mass) and cell membranes (muscle quality) within skeletal muscle ^(38)^. Consequently, it is suggested that when sarcopenia is combined with obesity, changes in SMM and SMQ are suppressed. Therefore, on the basis of the results of this study and previous studies, it can be inferred that chronic inflammation due to sarcopenic obesity in patients with stroke has a negative effect on changes in SMM and SMQ. A study investigating sarcopenic obesity in patients with stroke reported an association with physical function, SMM, and outcomes ^(5), (6), (25)^. Although some authors have examined sarcopenic obesity, its relationship with changes in SMM and SMQ over time has not been investigated, and few reports on the relationship between sarcopenic obesity and SMM and SMQ in patients with stroke exist, generating the lack of evidence. The present study has clarified the relationship between sarcopenic obesity diagnosed using the JWGS criteria and changes in SMM and SMQ, thereby helping elucidate sarcopenic obesity in patients with stroke. However, the data evaluated in this study did not allow a detailed investigation of inflammatory mediators related to obesity. Therefore, it is necessary to clarify this issue through further studies.

This research provides important clinical findings for patients with stroke who undergo rehabilitation. Systematic reviews and meta-analyses have shown that resistance training is effective in reducing inflammatory mediators and body fat in patients with sarcopenia and sarcopenic obesity ^(39), (40)^. Moreover, it has been shown that aerobic exercise for patients with overweight and obesity contributes to a decrease in factors related to obesity, such as total cholesterol, triglycerides, and low-density lipoprotein ^(41), (42)^. Resistance training and aerobic exercise are incorporated into rehabilitation programs for patients with stroke and are used to help restore physical function and ADL. Therefore, it is important to recommend a rehabilitation program that focuses on mitigating sarcopenia and obesity in patients with stroke with sarcopenic obesity.

This study has several limitations. First, this was a retrospective observational study conducted at a single center, and there is a possibility that confounding factors were not fully investigated. In addition, only a limited number of patients with stroke with sarcopenic obesity were included in this study, limiting the generalizability of the results. It is necessary to conduct future clinical research involving multiple facilities. Second, the statistical methods used in this study do not allow establishment of causal relationships between sarcopenic obesity and changes in SMM and SMQ. Therefore, it is necessary to investigate this issue through prospective validation in the future. Third, the results suggest that differences in rehabilitation programs affect SMM and quality. In this study, all patients received conventional rehabilitation from the day of admission. The rehabilitation program included standing, walking, and ADL exercises. However, the individual implementation of these exercises, such as frequency and intensity, was unclear and not standardized. Fourth, in this study, baseline neurological severity varied among patients, which may have affected the outcome. Therefore, it is necessary to clarify the impact of these differences in the future using a more rigorous research design with matched baseline patient characteristics.

In conclusion, the results of this study reveal that sarcopenic obesity is negatively associated with changes in SMM and SMQ in patients with stroke who undergo rehabilitation. This finding suggests that it is important to evaluate sarcopenic obesity in patients with stroke from an early stage.

## Article Information

### Conflicts of Interest

None

### Acknowledgement

We thank all patients who agreed to participate in this study.

### Author Contributions

Ryo Shiraishi: Data curation, formal analysis, investigation, methods, project administration, resources, supervision, and writing―original draft preparation. Nami Shiraishi: Resources, writing―reviewing and editing. Haruhiko Sato: Resources, writing―reviewing and editing. Takuya Tanaka: Resources, writing―reviewing and editing. Keita Shimizu: Resources, writing―reviewing and editing. Kota Okumura: Resources, writing―reviewing and editing. Kou Suzuki: Resources, writing―reviewing and editing. Takahiro Ogawa: Writing―reviewing and editing, supervision, project administration.

### Data Availability

The datasets generated and/or analyzed during the present study are available from the corresponding author on reasonable request.
